# Comprehensive determination of transcription start sites derived from all RNA polymerases using ReCappable-seq

**DOI:** 10.1101/gr.275784.121

**Published:** 2022-01

**Authors:** Bo Yan, George Tzertzinis, Ira Schildkraut, Laurence Ettwiller

**Affiliations:** 1 New England Biolabs Incorporated, Ipswich, Massachusetts 01938, USA

## Abstract

Determination of eukaryotic transcription start sites (TSSs) has been based on methods that require the cap structure at the 5′ end of transcripts derived from Pol II RNA polymerase. Consequently, these methods do not reveal TSSs derived from the other RNA polymerases that also play critical roles in various cell functions. To address this limitation, we developed ReCappable-seq, which comprehensively identifies TSS for both Pol II and non–Pol II transcripts at single-nucleotide resolution. The method relies on specific enzymatic exchange of 5′ m^7^G caps and 5′ triphosphates with a selectable tag. When applied to human transcriptomes, ReCappable-seq identifies Pol II TSSs that are in agreement with orthogonal methods such as CAGE. Additionally, ReCappable-seq reveals a rich landscape of TSSs associated with Pol III transcripts that have not previously been amenable to study at genome-wide scale. Novel TSS from non–Pol II transcription can be located in the nuclear and mitochondrial genomes. ReCappable-seq interrogates the regulatory landscape of coding and noncoding RNA concurrently and enables the classification of epigenetic profiles associated with Pol II and non–Pol II TSS.

Current widely used methods to characterize transcriptomes such as whole-transcriptome shotgun sequencing (RNA-seq) (Morin et al. 2008) fall short in providing accurate descriptions of transcriptional landmarks such as transcription start sites (TSSs), termination sites, and isoform composition. The identification of TSSs is essential to study gene regulation because it permits the association between RNA transcription and the underlying genomic landmarks such as promoters and histone marks. Alternative TSSs have been detected in >50% of human genes (Breitbart et al. 1987), driving most of the transcript isoforms differences across tissues (Reyes and Huber 2018). Genomic positions of alternative TSSs are often found within other isoforms of the same gene, confounding their detection using conventional RNA-seq.

Currently methods for TSS determination such as cap analysis of gene expression (CAGE) (Kodzius et al. 2006; Murata et al. 2014), NanoCAGE (Ivanchenko and Megraw 2018), and Oligo-capping (Maruyama and Sugano 1994) are limited to identifying 7mG capped polymerase (Pol) II–derived TSS. These methods entirely exclude the large number of TSSs derived from eukaryotic RNA Pol I, RNA Pol III, and mitochondrial RNA polymerase (POLRMT), which produce uncapped noncoding RNA. These uncapped primary transcripts display a 5′ triphosphate identical to the 5′ end of prokaryotic primary transcripts. With the growing body of literature highlighting the key role of both Pol III– and Pol II–transcribed noncoding RNA in regulating biological processes and diseases (Marshall and White 2008; Lekka and Hall 2018; Täuber et al. 2019), a method that comprehensively identifies TSS for all eukaryotic RNA polymerases would be consequential.

We have previously developed Cappable-seq to identify TSS in prokaryotic species (Ettwiller et al. 2016). Cappable-seq is based on the ability of the vaccinia capping enzyme (VCE) to add a biotinylated guanosine to 5′ di- or triphosphorylated RNA ends and streptavidin enrichment of those fragments. Building on Cappable-seq, we have developed ReCappable-seq to also capture 7-methyl G–capped transcripts derived from Pol II RNA polymerase. To achieve this, we took advantage of the property of the yeast scavenger decapping enzyme (yDcpS) to convert capped RNA (Liu et al. 2002) into di-phosphorylated RNA that can be “recapped” by the VCE (Wulf et al. 2019). Thus, ReCappable-seq enables the identification of TSS for RNA transcripts derived from all RNA polymerases.

The comparison of two data sets, one derived from RNA treated with calf intestinal alkaline phosphatase (CIP) and the other not, permits the discrimination between capped 5′ ends and triphosphorylated 5′ ends. Each TSS can be inferred as derived from either Pol II or non–Pol II polymerases, because Pol II transcripts are capped and not depleted with CIP, whereas non–Pol II transcripts are triphosphorylated and depleted with CIP.

We applied ReCappable-seq to the transcriptome of the A549 human cancer cell line with the aims to (1) identify all TSSs irrespective of their transcribing polymerase and (2) classify them into Pol II and non–Pol II TSS. We also performed CAGE on the same cell line in order to benchmark ReCappable-seq for the identification of Pol II TSSs.

## Results

### Preparation of ReCappable-seq libraries

**Figure 1.**
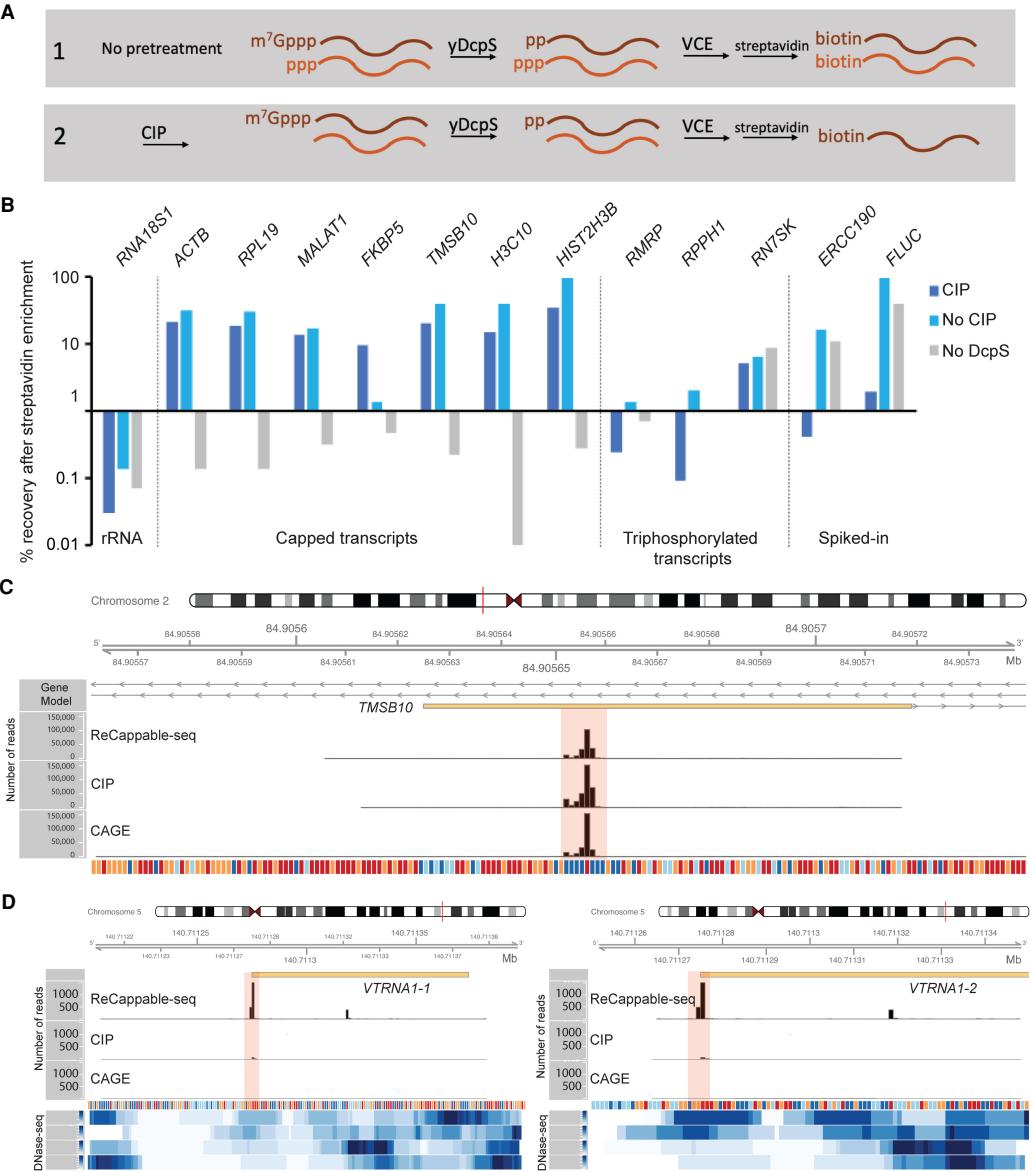
ReCappable-seq. (*A*) Principle of ReCappable-seq. (*1*) RNA is subjected to decapping with yDcpS, which acts on capped transcripts originating from Pol II transcription. Subsequently, the RNA is capped with a biotin-modified GTP analog (3′-desthiobiotin-GTP) using VCE. This biotinylation step allows enrichment of all primary transcripts on a streptavidin matrix. (*2*) Differentiation of Pol II from the non–Pol II transcripts is accomplished by sequencing a second library constructed with RNA treated with CIP before the yDcpS treatment, in order to remove the 5′ triphosphate from non–Pol II transcripts. (*B*) RT-qPCR assay measuring the recovery after streptavidin enrichment of various classes of transcripts such as *RNA18S1* rRNA as an example of a processed transcript; *ACTB*, *RPL19*, *MALAT1*, *FKBP5*, *TMSB10*, *H3C10*, and *HIST2H3B* as examples of capped transcripts; *RMRP*, *RPPH1*, and *RN7SK* as examples of Pol III transcripts (with *RN7SK* having a 5′ methylated triphosphate and therefore being resistant to CIP treatment; see main text); and *ERCC190* and *FLUC* as examples of spiked-in in vitro transcripts with a defined triphosphorylated 5′ end. The Cq values are available in Supplemental Figure S1F. (*C*) Example of a Pol II TSS in the *TMSB10* locus: The same positions (shaded in pink) are found in the CAGE data set. CIP treatment intensifies the signal, consistent with a Pol II TSS. (*D*) Example of Pol III TSS corresponding to the start of two vault RNAs (*vtRNA1-1* and *vtRNA1-2*) located on Chr 5. The positions (shaded in pink) are missing in the CAGE data set. CIP treatment reduces the signal, consistent with non–Pol II TSS. In *C* and *D*, the tracks correspond to ReCappable-seq, CIP-ReCappable-seq, and CAGE read coverage (number of reads). All libraries were down-sampled to the same number of total mapped reads (63,300,000) to facilitate comparison. The four *bottom* tracks correspond to read density from public ENCODE DNase-seq from A549 cells (ENCFF473YHH, ENCFF809KIH, ENCFF821UUL, ENCFF961WXW).

The principle of ReCappable-seq (shown schematically in Fig. 1A) relies on the tagging of all primary transcripts with biotin. RNA is subjected to decapping with yDcpS, which acts on capped transcripts originating from Pol II transcription. yDcpS hydrolyzes the phosphodiester bond between the gamma and beta phosphates of the 7mG-cap (7mGppp-RNA), leaving a diphosphate end (Wulf et al. 2019). Importantly, cap0 and cap1, as well as m7Gpppm6A and m7Gpppm6Am, are all substrates for yDcpS (Wulf et al. 2019). Subsequently, the RNA is capped with a biotin-modified GTP analog (3′-desthiobiotin-GTP) using VCE. At this step, the decapped diphosphate RNA (pp-RNA) product of yDcpS and the 5′ triphosphorylated RNA (ppp-RNA) originating from Pol I, Pol III, and POLRMT (non–Pol II) transcription are capped with the biotin-modified analog. This biotinylation step allows enrichment of all primary transcripts on a streptavidin matrix (Fig. 1A). Differentiation of Pol II from the non–Pol II transcripts is accomplished by sequencing a second library constructed with RNA treated with CIP before the yDcpS treatment, in order to remove the 5′ triphosphate from non–Pol II transcripts (Fig. 1A). This treatment prevents the non–Pol II transcripts from being capped with biotin and enriched. The enriched RNA from each RNA sample is subsequently decapped with RppH to generate 5′ monophosphate ends, which are captured in a ligation-based library. Comparison of these two libraries allows the distinction of capped transcripts from 5′ triphosphate transcripts.

### Validation of the ReCappable-seq principle by RT-qPCR

To show that primary transcripts bearing either a 5′ cap or a 5′ triphosphate can be specifically captured from total RNA, we subjected total RNA from A549 cells to the enzymatic and streptavidin steps described in Figure 1A and quantified specific transcripts using RT-qPCR. To show the requirement of the decapping step, we also processed samples for which the yDcpS step was omitted.

The results showed substantial recovery of both Pol II and Pol III primary transcripts after streptavidin enrichment, whereas 18S ribosomal RNA (*RNA18S1*), a noncapped, nontriphosphorylated transcript, was significantly depleted (Fig. 1B). As expected, when the decapping step was omitted, capped transcripts were depleted, whereas triphosphorylated Pol III transcripts were recovered (Fig. 1B). Conversely, when RNA was CIP treated, capped transcripts were recovered, whereas triphosphorylated Pol III transcripts were depleted (Fig. 1B). An exception is *RN7SK*, known to possess a monomethyl gamma-phosphate at its 5′ end (Cosgrove et al. 2012), which is resistant to CIP (Gupta et al. 1990). These results show that the yDcpS decapping and VCE recapping steps specifically allow the recovery of capped transcripts and that CIP treatment enables the distinction of m^7^G-capped RNA from triphosphorylated RNAs (Fig. 1B).

### Genome-wide identification of TSSs using ReCappable-seq

We applied ReCappable-seq to total RNA isolated from the human cell line A549. Two samples (untreated and pretreated with CIP) were processed using the steps outlined in Figure 1A. The RNA was then ligated with adaptors to prepare libraries suitable for short-read high-throughput sequencing as previously described by Ettwiller et al. (2016) (for protocol details, see Methods). This strategy results in the genome-wide identification of TSSs derived from all RNA polymerases at single-nucleotide resolution.

To evaluate the reproducibility of ReCappable-seq, we used technical replicates yielding approximately 32 million single-end Illumina reads per library (Supplemental Table S1). In addition, for both the CIP-treated and untreated samples, unenriched libraries were constructed for each from an aliquot taken before the streptavidin enrichment step. Reads were mapped to the human genome using STAR with the ENCODE default parameters (Methods) (Dobin et al. 2013).

In parallel, we used RNA from the same A549 RNA preparation and performed CAGE for comparison. Analysis of the ReCappable-seq technical replicates at single-nucleotide resolution reveals a high correlation (Pearson corr = 0.96, *P*-value < 2.2 × 10^−16^) between replicates (Supplemental Fig. S1A), showing high reproducibility of the technique. ReCappable-seq replicates were combined and down-sampled to 63 million mappable reads for subsequent analyses.

We evaluated the specificity of ReCappable-seq for primary transcripts (5′ m^7^G-capped or 5′ triphosphorylated ends) versus nonprimary transcripts, using the fraction of reads mapping to rRNA as a surrogate for nonprimary transcripts. rRNAs are formed by the processing of a single pre-rRNA 45S transcript to form the mature 18S, 5.8S, and 28S rRNAs (Financsek et al. 1982). rRNAs account for the vast majority of the RNA mass in the cell, and because they are processed, they are expected to be depleted in ReCappable-seq libraries. Accordingly, the percentage of rRNA mapped reads drops from ∼70% in the unenriched control libraries to 3%–4% in the ReCappable-seq libraries (Supplemental Fig. S2A; Supplemental Table S1), highlighting the specificity of ReCappable-seq and its efficiency in removing transcripts with processed or degraded 5′ ends.

Mapped reads are found near the 5′ end of both protein-coding transcripts (Supplemental Fig. S2B) as well as noncoding transcripts known to be transcribed by Pol III (for an example, see Fig. 1C,D), suggesting high specificity for both types of TSSs. To further investigate the specificity of ReCappable-seq for primary 5′ ends, we tested ReCappable-seq on prefragmented RNA samples (RIN number < 3) prepared by magnesium ion–mediated fragmentation to simulate naturally occurring RNA degradation in biological samples. RNA degradation has been proven to be challenging for the determination of TSS because the majority of the 5′ ends are generated from fragments and do not correspond to TSSs.

The profile of mapped reads shows a high correlation between both prefragmented replicates (Pearson corr = 0.97) (Supplemental Fig. S1C) and reasonable correlation between prefragmented and intact starting material (Pearson corr = 0.76) (Supplemental Fig. S2C). Furthermore, reads from prefragmented material predominantly map to the start of annotated genes consistent with the positioning of TSSs (Supplemental Fig. S2B,D). Importantly, the percentage of reads mapping to processed rRNA drops from 65% in the unenriched control libraries to ∼1.3% in the ReCappable-seq libraries derived from prefragmented RNA (Supplemental Fig. S2A). Thus, ReCappable-seq is not affected by the large excess of uncapped 5′ ends resulting from fragmentation, and genuine primary 5′ ends were predominant in the ReCappable-seq libraries. Together, these results show that ReCappable-seq performs well on intact and fragmented RNA, adding further support to its high specificity for identifying 5′ ends of primary transcripts.

### Classification of TSSs into Pol II and non–Pol II TSS

Candidate TSSs were identified and quantified at single-nucleotide resolution using a TSS analysis pipeline (Methods). In short, candidate TSSs were defined as single-nucleotide positions in the genome where the number of 5′ ends of the reads mapping to those is above 1 TSS tags per million mapped reads (TRM). From 63 million primary mappable reads, a total of 42,988 candidate TSSs were identified genome-wide.

**Figure 2.**
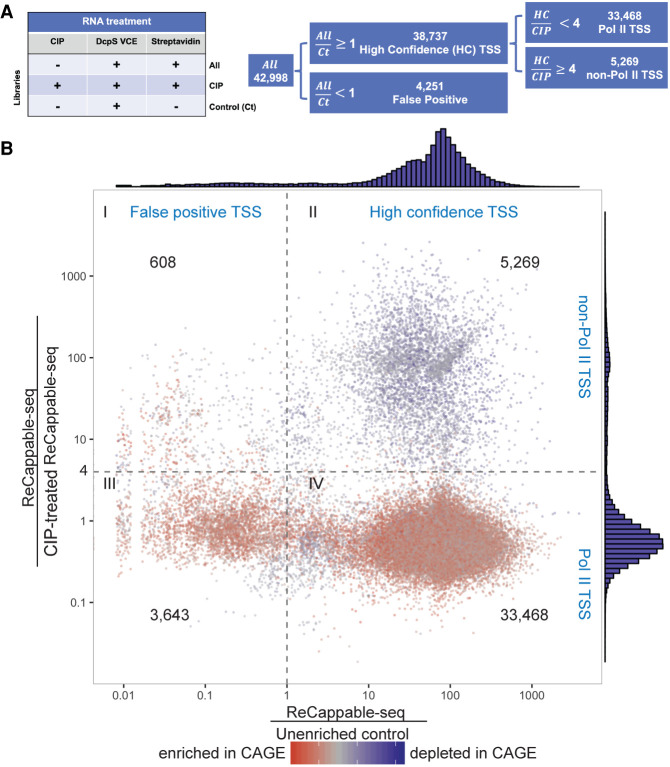
Overview of the sequencing result. (*A*) Summary of sequencing experiments: Experiments were performed, resulting in three data set types: ReCappable-seq (ALL), CIP-treated ReCappable-seq (CIP), and a unenriched control for which the streptavidin step has been omitted (Ct). ALL corresponds to the ReCappable-seq data set without CIP treatment and defines 42,988 candidate TSSs. Comparison of ALL with Ct enables the definition of 38,737 high-confidence (HC) TSSs. Comparison of HC with CIP enables the definition of HC Pol II TSSs (33,468) and the HC non–Pol II TSSs (5269). (*B*) Candidate TSS positions (42,988) are distributed according to the TPM ratio between ALL and Ct (*x*-axis) and the TPM ratio between ALL and CIP (*y*-axis). Dotted lines define four quadrants as follows: ALL ÷ CIP less than four (Pol II TSS), ALL ÷ CIP above or equal to four (non–Pol II TSS), ALL ÷ Ct above or equal to one (HC TSS), and ALL ÷ Ct less than one (false-positive TSS). Colors denote the ratio between ALL and CAGE with red (enriched in CAGE relative to ReCappable-seq) and blue (depleted in CAGE relative to ReCappable-seq).

To evaluate the specificity of ReCappable-seq in capturing capped and triphosphate RNAs, an enrichment score was calculated for each candidate TSS by dividing the TPM in the ReCappable-seq libraries with the TPM in the unenriched control library (Ct) (Fig. 2A). Candidate TSSs depleted in the ReCappable-seq library (ratio less than one) are considered false positives (Fig. 2B, quadrants I and III). The enriched candidate TSSs (ratio equal to or greater than one) are considered high-confidence TSSs (Fig. 2B, quadrants II and IV); 38,737 of the 42,988 candidate TSSs were high confidence, indicating that the majority (90.1%) of the TSS identified by ReCappable-seq are true positives. Unless otherwise stated below, the term TSS refers to the high-confidence TSSs.

To enable the discrimination of TSS derived from capped transcripts from those derived from triphosphate transcripts, we used the data from RNA pretreated with CIP before performing ReCappable-seq (Fig. 1A; Supplemental Fig. S1B). Comparison of the non-CIP ReCappable-seq data set with the CIP-treated data set reveals two populations composed of 33,468 CIP-resistant TSSs consistent with Pol II transcripts and 5269 CIP-sensitive TSSs consistent with non–Pol II transcripts (Fig. 2B, quadrants II and IV, respectively).

### TSSs consistent with Pol II transcripts

We validated Pol II–consistent TSSs from ReCappable-seq using data from orthogonal methods known to mark TSSs, such as CAGE and publically available data for DNase-seq, chromatin immunoprecipitation (ChIP)–seq for Pol II, and manually curated human gene annotation. For CAGE, we used data sets derived from the same A549 RNA preparation used for ReCappable-seq.

**Figure 3.**
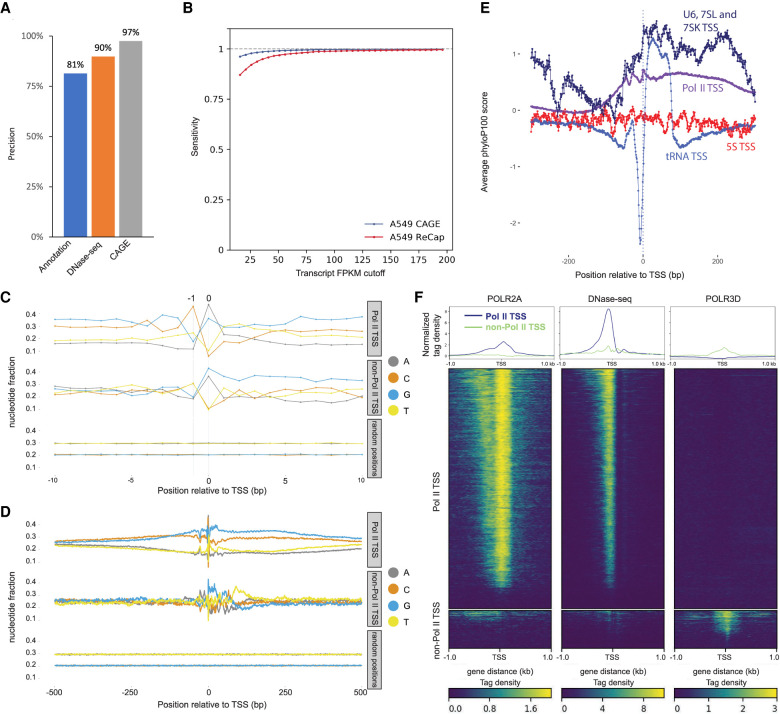
TSS Characterization. (*A*) Precision (TP/(TP + FP)) × 100 of ReCappable-seq. (TP) True positive, (FP) false positive (see Methods). (*B*) Sensitivity (TP/(TP + FN)) of ReCappable-seq. (TP) True positive (ReCappable-seq TSS with UCSC annotation), (FP) false positive (TSSs in UCSC annotation but not detected by ReCappable-seq; see Methods). (*C*) Nucleotide composition in the 20-bp flanking region for Pol II TSSs (*top*), non–Pol II TSSs (*middle*), and randomized genomic positions (*bottom*). (*D*) Same as *C* for 1-kb flanking region; 83.7% and 79.5% of Pol II and non–Pol II TSSs, respectively, start with *A* or *G*. (*E*) Conservation profiles using phyloP basewise conservation score (Pollard et al. 2010) derived from MULTIZ alignment (Blanchette et al. 2004) of 100 vertebrate species around Pol II TSSs (purple), tRNA TSSs (light blue), 5S TSSs (red), and U6, 7SL, and 7SK TSSs (dark blue). (*F*) Profiles and heatmaps of Pol II (*left* panel) and Pol III (*right* panel) ChIP-seq and DNase-seq (*middle* panel) at Pol II TSSs and non–Pol II TSSs. The Pol II ChIP-seq and DNase-seq data have been downloaded from the ENCODE website (Davis et al. 2018); the Pol III ChIP-seq data have been obtained from Oler et al. (2010).

We assessed precision and sensitivity of ReCappable-seq following the published protocol previously described (Adiconis et al. 2018). We compared ReCappable-seq with CAGE and found that 97% of the ReCappable-seq TSSs have a CAGE signal within a window of ±1 nucleotide (nt) (Fig. 3A). We found, when compared against gene annotation and DNase-seq, precision of 81% and 90%, respectively (Fig. 3A). When CAGE is compared with DNase-seq sites and gene annotation, the accuracy is similar to that of ReCappable-seq. CAGE shows higher sensitivity, presumably owing to the fact that ReCappable-seq data have less sequence depth for Pol II TSSs because they include non–Pol II TSSs, accounting for ∼50% of the sequencing reads (Fig. 3B).

### Comparison between Pol II and non–Pol II TSSs

We analyzed the nucleotide composition for Pol II and non–Pol II TSSs and found that 83.7% and 79.5%, respectively, of the +1 nucleotides are A or G and 70.6% and 61.7%, respectively, of the −1 position are C or T (Fig. 3C,D), revealing the canonical −1[C or T] +1[G or A] motif typical for TSSs for both Pol II and non–Pol II TSSs.

We examined the conservation profiles around Pol II and the different types of Pol III TSSs for 7SL RNAs (7SL), 7SK snRNAs (7SK), U6 snRNAs (U6), tRNAs, and 5S rRNAs (5S) gene families. We found a peak of conservation for Pol II TSSs consistent with the fact that these sites are functional and therefore under selection (Fig. 3E). tRNA conservation profiles have a very distinct conservation profile found in the highly variable region upstream of the TSSs. This region has been shown to experience elevated mutation rates (Thornlow et al. 2018), reflecting very different evolutionary pressures compared with the other classes of transcripts (Fig. 3E).

Using data from ENCODE Pol II ChIP-seq from A549 cells and Pol III ChIP-seq from HeLa cells (the only Pol III ChIP-seq data publicly available) (Oler et al. 2010), we interrogated the binding profiles of both RNA polymerases relative to ReCappable-seq-derived Pol II and non–Pol II TSSs. Because the Pol III ChIP-seq data are from a different cell line, concurrence of Pol III polymerase binding sites with TSSs may only be found for commonly expressed genes between HeLa and A549, and an absence of a ChIP-seq signal is inconclusive. Consistent with the origin of Pol II and non–Pol II TSSs, we found a higher density of Pol II ChIP tags (Oler et al. 2010) around Pol II TSSs (Fig. 3F) and a higher density of Pol III ChIP tags around non–Pol II TSSs (Fig. 3F). It has been shown that the chromatin landscape of Pol III–transcribed genes resembles that of Pol II–transcribed genes, although there are clear differences (Barski et al. 2010; White 2011). Substantial differences in chromatin structure of Pol II and Pol III promoters have been reported (Helbo et al. 2017). With the ability to identify and classify Pol II and non–Pol II consistent TSSs, we studied the differential positioning of chromatin marks and DNA-interacting proteins relative to both TSS classes. For this, we compared ReCappable-seq TSSs with published ChIP-seq from ENCODE data performed on A549 cells to identify potentially interesting differential chromatin marks associated with Pol II and non–Pol II consistent TSSs.

Most of the chromatin landscape around TSSs of non–Pol II–transcribed genes resembles that of Pol II–transcribed genes in agreement with the literature (Supplemental Fig. S3A; Barski et al. 2010; White 2011). Nonetheless, we find examples of specific transcription factor (TF) binding profiles at non–Pol II–transcribed genes that have not been previously reported to be associated with the regulation of non–Pol II genes (Supplemental Text 1; Supplemental Fig. S3A). For example, SREBF2 (also known as SREBP2), a ubiquitously expressed TF known to control cholesterol homeostasis (Horton et al. 2002), is binding almost exclusively at non–Pol II TSSs. This result strongly suggests a role of SREBF2 in the regulation of non–Pol II–transcribed genes.

Distinct DNase I accessibility profiles can be observed at Pol II versus non–Pol II TSSs. Pol II transcripts show maximum DNase I accessibility a few nucleotides upstream of their TSSs (Fig. 3F) consistent with nucleosome depletion at Pol II TSSs (Radman-Livaja and Rando 2010). In contrast, non–Pol II transcripts show minimal DNase I accessibility at the TSSs (Fig. 3F). Because there are three types of Pol III promoters, we further grouped the non–Pol II TSSs into three categories—type I (5S), type II (tRNA), and type 3 (U6, 7SK, and 7SL); identified the DNase I footprints for each group; and plotted the DNase I accessibility profiles for each type separately. We observed differences in DNase I sensitivity between the different classes (Supplemental Fig. S3B). This result is in accordance with previous findings that show sharp nucleosome positioning differences between tRNA genes and other Pol III genes (Helbo et al. 2017). Our results further extend the distinctive accessibility of DNA at the TSSs of Pol II and non–Pol II transcripts and highlight a distinct chromatin landscape for Pol II and non–Pol II TSSs.

**Figure 4.**
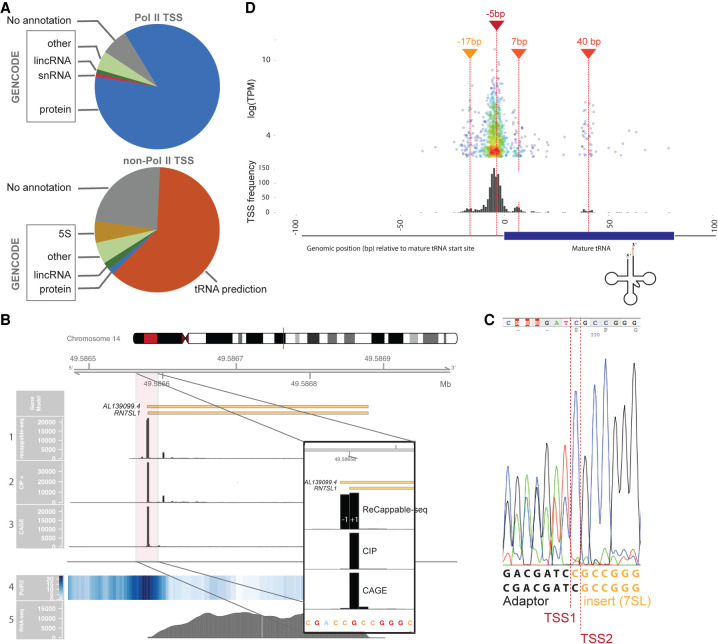
Pol II and non–Pol II TSS distribution. (*A*) Pie charts representing the proportion of TSSs associated with GENCODE genes, associated with predicted tRNAs (orange), or not associated with any annotation (gray) for Pol II (*upper* chart) and non–Pol II (*lower* chart). (*B*) *RN7SL1* locus showing the distribution of the 5′ end of mapped reads for ReCappable-seq (track 1), CIP-treated ReCappable-seq (track 2), CAGE (track 3), Pol II ChIP-seq (track 4, read density), and RNA-seq reads (track 5). Libraries in tracks 1, 2, and 3 were down-sampled to the same number of total mapped reads. The floating panel represents a close up of the 5′ end of the *RN7SL1* with two TSS at −1 and +1 of the annotated *RN7SL1*. The 5′ mapped end of the reads is shown for ReCappable-seq (track 1), CIP-treated ReCappable-seq (track 2), and CAGE (track 3) to mark the TSS positions. (*C*) Validation of the two TSSs identified for *RN7SL1* using RACE. The amplified fragments were directly sequenced using Sanger sequencing with a primer located in the *RN7SL1* gene (Methods). The sequencing trace reveals two products ligated to the RACE adaptor resulting from two alternative transcript starts corresponding to TSS1 and TSS2. (*D*) Non–Pol II TSSs flanking tRNA annotations. The *top* panel visualizes individual non–Pol II TSSs relative to the 5′ end of the annotated mature tRNA (in bp) as a function of the TPM. The *bottom* panel represents the distribution of the non–Pol II TSSs relative to the start of the annotated tRNA starts (in bp).

Pol II TSSs were found for 7186 annotated genes. Consistent with the function of Pol II polymerase, the majority (89%) of ReCappable-seq Pol II TSSs are found either upstream or within protein-coding genes (Fig. 4A). Additionally, because enhancer-associated Pol II transcription (eRNA) has been reported (Mikhaylichenko et al. 2018), we examined the profile of ReCappable-seq reads within 10 kb of enhancers in A549 (Methods) (Gao and Qian 2020). Most of these ReCappable-seq reads are located within 500 bp upstream of or downstream from the enhancer centers (Supplemental Fig. S4A,B). This configuration may lead to bidirectionally convergent transcribed enhancers as exemplified in Supplemental Figure S4C or divergent TSSs as exemplified in Supplemental Figure S4D and E. We found a total of 5612 and 1718 Pol II and non–Pol II TSSs, respectively, overlapping A549 enhancers.

### Non–Pol II TSSs

Non–Pol II TSSs account for 5269 positions. In addition to the correlation of the TSSs to Pol III ChIP-seq (Fig. 3F), we validated some non–Pol II consistent TSSs using 5′ RACE (Supplemental Fig. S5). The 5′ RACE results for two uniquely mapped annotated Pol III–transcribed genes (*RMRP* and *RPPH1*) confirmed that the start positions were the same as the major TSSs detected from the ReCappable-seq data.

The 5269 non–Pol II TSSs (TPM ≥ 1) represent ∼50% of the total reads despite representing only 14% of all TSS positions, consistent with the fact that Pol I and Pol III transcripts are abundant. Of these non–Pol II TSSs, 4032 are detected within or upstream of 758 genes, of which 314 are annotated as Pol III–transcribed genes such as *RMRP* and *RPPH1*, 5S RNA, Vault RNAs, 7SL, and YRNA gene families. The other 444 are located at predicted tRNA genes (Fig. 4A; Supplemental Fig. S6; Chan and Lowe 2016). The remaining 1237 detected TSSs correspond to neither GENCODE annotation nor tRNA predictions by tRNAscan and are distributed in approximately 700 loci (Methods).

Around one-third of the non–Pol II TSSs (34%) are derived from reads mapping to multiple positions in the genome, consistent with the role of Pol III polymerase in transcribing repeat elements and multiple-copy genes (White 2011) such as 5S, 7SK, and 7SL (Canella et al. 2010). We therefore proceeded with a genome-wide investigation of non–Pol II TSSs with respect to such genes and located them upstream of or at the starts of 5S, 7SL, tRNA, and U6 genes and HY and MIR repeats (Supplemental Fig. S7A). This result is in agreement with literature that has assigned transcription of these genes to Pol III (White 2011). A closer look at the *RN7SL1* gene (a member of the 7SL gene family) identifies two strong TSSs at +1 and −1 bp relative to the annotated gene start (Fig. 4B). The −1 TSS is mostly eliminated by CIP treatment, consistent with the triphosphorylated nature of the 5′ end. Conversely, the +1 TSS is not affected by CIP treatment, consistent with a possible canonical cap structure at the 5′ end of 7SL. We experimentally confirmed the presence of both TSSs using 5′ RACE followed by Sanger sequencing (Methods) (Fig. 4C). The occurrence of a canonical cap structure at +1 of *RN7SL1* would sharply contrast with the body of literature that describes 7SL genes as being transcribed by Pol III (Canella et al. 2010). Alternatively, the 5′ end of the +1 TSS form may not be accessible to CIP treatment, leading to its incorrect assignment to Pol II. However, in further support of a Pol II TSS, we also observe a CAGE signal.

#### 7SK *gene family*

The TSSs identified at the start of 7SK genes appear resistant to CIP treatment and, as such, were classified as Pol II TSSs (Supplemental Fig. S7A). Consistently, RT-qPCR results showed that neither CIP nor yDcpS treatment affects the recovery of 7SK upon streptavidin enrichment (Fig. 1B). It has previously been shown that the 5′ end of the 7SK transcripts contains a monomethyl gamma-phosphate on the 5′ triphosphate (Cosgrove et al. 2012), conferring resistance to alkaline phosphatase (Gupta et al. 1990). We have confirmed this experimentally (Methods) (Supplemental Fig. S7B). Furthermore, we show that VCE can remove the gamma-methylated phosphate, indicating that the RNA triphosphatase activity of VCE is not blocked. This suggests that such 5′ ends are subject to in vitro capping (Supplemental Methods; Supplemental Fig. S7B). Together, these results explain why 7SK TSSs are classified as non–Pol II (CIP resistant and not dependent on yDcpS decapping.)

#### *U6 sn*RNA *gene family*

The same result should have been obtained for the U6 small nuclear RNA, which is also known to possess a gamma-methyl triphosphorylated 5′ end (Singh and Reddy 1989; Didychuk et al. 2018). However, we found no TSS signal from the U6 genes at the annotated start; instead, we found CIP-sensitive TSSs ∼20 bp upstream (Supplemental Fig. S7A), consistent with a nonmethylated canonical triphosphate RNA 5′ end originating at these positions. To rule out an uncharacteristic cell line–specific expression of U6 in A549, we investigated U6 TSSs in data from ReCappable-seq performed on human brain RNA.

Because U6 genes are highly repeated, we modified the parameters for multiple mapping, allowing reads to map to more loci than the default ENCODE mapping parameters. TSS signals at the 5′ end of U6-annotated genes can be found for both A549 and the human brain (see Supplemental Text 1; Supplemental Fig. S7C). Nonetheless, TSSs at annotated U6 starts are derived from very few reads in both samples, inconsistent with the abundance of U6 (Montzka and Steitz 1988).

To further explore the U6 TSS findings, we performed 5′ RACE on total A549 RNA using a complementary internal U6 primer (Methods). After amplification and sequencing of the resulting cDNA, we found evidence for both the annotated and the upstream TSSs (Supplemental Fig. S7C). We did not recover a substantial amount of the expected U6 transcripts, because of either secondary structure or some other unknown feature of the RNA that prevents capture by our enrichment and cloning protocol.

#### *t*RNA*s*

Pre-tRNAs have been notoriously difficult to study owing to the rapid processing of primary transcripts relative to the exceptional stability of the mature tRNA, which consequently accumulates in the cell (Wilusz 2015). With the ability of ReCappable-seq to capture primary transcripts, we are now in a unique position to interrogate the TSS landscape of tRNAs. Using annotated tRNAs from GtRNAdb (Chan and Lowe 2016), we found 3245 of the non–Pol II TSSs are located upstream or within 444 tRNA genes representing the largest class of non–Pol II TSSs found by ReCappable-seq (Fig. 4D; Supplemental Fig. S6). Notably *tRNA-Leu-TAG-1-1* is the highest expressed tRNA (TPM 531,81), accounting for ∼10% of the total non–Pol II reads in A549 cells.

TSS positions relative to the tRNA annotation highlight a large number of TSSs ∼5 bp upstream of mature tRNA 5′ ends (Fig. 4D), consistent with previous work using in vitro transcription, which identified TSSs located mostly 10 to 2 bp upstream of mature plant tRNAs (Yukawa et al. 2011). Additionally, we found three novel TSS clusters at around −17, +7, and +40 bp from the mature tRNA 5′ end (Fig. 4D). Further refinement relative to the type of tRNA reveals that downstream TSSs are mostly found in Leu tRNA and Lys tRNA (Supplemental Fig. S8).

#### Identification of the human mitochondrial TSSs

The property of ReCappable-seq to capture 5′ di- and triphosphorylated transcripts offers the unique ability to interrogate the TSS landscape of the mitochondrial genome (Supplemental Fig. S9A). The entire mitochondrial genome is known to be transcribed from both strands as long polycistronic transcripts (D'Souza and Minczuk 2018). The light-strand promoter (LSP) controls the transcription of eight of the tRNAs and the *MTND6P1* genes. On the heavy strand, a two-promoter system (HSP1 and HSP2) has historically been proposed to explain the higher abundance of the rRNAs. However, the two-promoter model remains controversial as more recent experiments (Litonin et al. 2010; Terzioglu et al. 2013) suggest that heavy-strand transcription is under the control of a single promoter (HSP1) and that the difference in abundance may be a consequence of differential turnover (D'Souza and Minczuk 2018).

In agreement with the literature, ReCappable-seq identifies LSP and HSP1 TSSs at nucleotide resolution. Both TSSs have a strong major peak and a few minor peaks indicative of an imprecise start of transcription (Supplemental Fig. S9B,C). Most of the transcripts starting at the major HSP1 TSSs show signs of stuttering with nontemplated adenosines at the start of the transcripts (Supplemental Fig. S9C). Although such stuttering has been reported previously during initiation of transcription by phage polymerases in vitro (Cunningham et al. 1991), the addition of nontemplated nucleotides in human mitochondria has not been previously described.

We did not find TSSs at the HSP2 position, reinforcing the absence of an HSP2 promoter as indicated by previous studies (Litonin et al. 2010; Terzioglu et al. 2013). Instead, we found three putative novel TSSs across the mitochondrial genome, two on the heavy strand (positions MT:2434 and MT:3242, human GRCh38) and one on the light strand (position MT:16029). The novel TSS on the light strand (Supplemental Fig. S9D) is located 6 bp upstream of the proline tRNAs and shows similar conformation to the HSP1 with stuttering of nontemplated adenosines at the start of the novel transcripts. Similar nucleotide composition starting at the TSS can be found for HSP1, LSP, and the predicted novel TSS (position MT:16029), with AAAGA as the common motif.

## Discussion

In this work, we describe a novel technology, ReCappable-seq, which identifies eukaryotic TSSs for all primary transcripts genome-wide at single-nucleotide resolution. Our method relies on decapping and recapping RNA with two sequential enzymatic treatments using the enzymes yDcpS and VCE, as well as subsequent enrichment with streptavidin. Starting with 2–5 µg of total RNA and using standard ligation-based Illumina library kits, without the need of custom adaptor/primers, one can obtain TSS information with very low ribosomal reads content. Compared with standard CAGE protocols (e.g., N-antiCAGE), our method requires less time, is technically less difficult to perform, uses fewer steps, does not require customized reagents, and should be more accessible to researchers.

ReCappable-seq identifies TSSs independently of the transcribing polymerases because it captures both capped and triphosphorylated primary transcripts. In this respect, ReCappable-seq represents a significant departure from existing technologies that either target prokaryotic TSSs (Sharma et al. 2010; Ettwiller et al. 2016) or eukaryotic Pol II TSSs (Adiconis et al. 2018). With the growing realization of the importance of noncoding RNA, the ability of ReCappable-seq to provide a comprehensive global landscape of TSSs is an important advance. Indeed, deregulation of Pol III–transcribed genes has been shown to be associated with a large variety of human disorders such as, but not limited to, cancer, neurodegenerative diseases, and autoimmune disease (Yeganeh and Hernandez 2020). For example, the changes of 5S rRNA, tRNA, or regulatory RNA (e.g., *RN7SK*) levels can lead to drastic changes in mRNA expression. The ability of ReCappable-seq to quantitatively measure the TSS usage of all transcripts irrespective of the transcribing polymerase will permit the inclusion of Pol I and III TSSs into transcriptome studies that have been, so far, either ignored or studied separately from Pol II transcripts.

Similarly, ReCappable-seq can be applied to complex communities composed of both prokaryotic and eukaryotic organisms with the expected outcome of detecting all TSSs regardless of the organism or transcribing polymerases while simultaneously depleting mature rRNA.

In this study, we focused on the human A549 transcriptome and showed that ReCappable-seq identifies TSSs from transcripts derived from Pol I, Pol II, Pol III, and POLRMT RNA polymerases. Additionally, we have performed ReCappable-seq on various other cell lines and tissues, which showed broad applicability and reproducibility of ReCappable-seq (Supplemental Figs. S1E, S10A). Notably, Pol II and non–Pol II TSSs were identified genome-wide at base resolution for Jurkat and K562 cells (Supplemental Fig. S10B–D).

ReCappable-seq is in good agreement with CAGE results and other orthogonal methods for TSS identification. We further confirm the specificity of ReCappable-seq for genuine TSSs by showing that the TSSs obtained from highly degraded samples are comparable to TSSs obtained using intact RNA. Because ReCappable-seq identifies all TSSs, including those from highly expressed Pol III transcripts, the sensitivity for scarce Pol II transcripts is lower than CAGE. To achieve equivalent sensitivity as CAGE for Pol II TSSs, it would be necessary to increase the sequencing depth of ReCappable-seq. Alternatively, if the focus of the work is exclusively on Pol II transcripts, then sensitivity can be increased by removing non–Pol II TSSs with CIP pretreatment of the RNA.

ReCappable-seq can be complemented with an unenriched control library to better discriminate between primary transcripts and 5′ ends of processed/degraded RNA, leading to the identification of high-confidence TSSs. However, these unenriched control libraries double the number of libraries to be sequenced. With an estimated <10% of false-positive TSS positions, this library is optional but is useful for studying highly expressed genes that tend to have more spurious TSSs. Using the data from the ReCappable-seq CIP library allows assignment to either Pol II or non–Pol II TSSs. Nonetheless, a definitive assignment to the transcribing polymerase may require further evidence, considering the potential presence of noncanonical caps that are resistant to yDcpS or RNA structures that are CIP resistant.

We have shown that this grouping of TSSs into two distinct classes based on the sensitivity to CIP treatment is accurate for transcripts with 7mG cap structures and 5′ triphosphate ends. ReCappable-seq identifies some transcripts that do not have a canonical structure at their 5′ end. We show, for example, that although *RN7SK* RNA is a known Pol III transcript, the transcripts of *RN7SK* appear CIP resistant. This disparity is owing to the CIP-resistant gamma-methylated triphosphate found at 5′ ends of *RN7SK* transcripts, and consequently, the *RN7SK* TSS is classified here as Pol II consistent. The Pol III–transcribed *RN7SL1* gene is an interesting example in which ReCappable-seq identifies two adjacent TSSs. The one corresponding to the annotated gene start is classified as Pol II and is also identified by CAGE (Fig. 4B). The other one located 1 nt upstream is classified as Pol III. Further investigation is required to uncover the exact nature of the *RN7SL1* 5′ end. ReCappable-seq recovered TSSs for the major known Pol III–transcribed genes at single-nucleotide resolution with the notable exception of the U6 snRNA covered by only a few reads. It is possible that some RNA modification and/or RNA structure may affect the ability of ReCappable-seq to capture or sequence some transcripts as may be the case for U6. This may also be the case for tRNA molecules that have been shown to be heavily modified (Pan 2018) with some of the modifications blocking the cDNA synthesis step. Because our enrichment strategy relies on the 5′ triphosphate end, we recover primary tRNA transcripts before processing and modification.

Beyond the *RN7SL1* and *RN7SK* examples, the systematic complementation of ReCappable-seq results with orthogonal data sets such as CAGE can be used as a discovery platform to identify other interesting noncanonical capped structures. Indeed, a number of noncanonical cap structures recently reported (Abdelhamid et al. 2014; Bird et al. 2018) are expected to have distinct outcomes. For example, the trimethyl G cap is expected to be resistant to yDcpS (Wulf et al. 2019) but captured by CAGE, leading to a discrepancy between ReCappable-seq and CAGE. Despite trimethyl G cap having been described on U1, U2, U4, and U5 snRNAs (Shuman 2007), ReCappable-seq identifies a clear Pol II consistent TSS at the start of these genes, suggesting that perhaps only a fraction of the 5′ end of these transcripts has been fully methylated to trimethyl G.

NAD cap is another structure that will not be decapped by yDcpS (Wulf et al. 2019) but captured by CAGE because of the presence of the 2′,3′ diol on the ribose. As an example, the HSP1 TSS in the mitochondria has a strong CAGE signal consistent with the NAD cap recently reported in human mitochondria (Bird et al. 2018), whereas ReCappable-seq shows a CIP-sensitive signal consistent with a triphosphate end. Taken together, these results suggest a mixed population (NAD caps and triphosphates) at the 5′ end of RNA transcripts, which is initiated at the HSP1 TSS position.

Finally, with the ability to identify the 5′ ends of primary transcripts initiated by all the RNA polymerases, ReCappable-seq reveals a rich landscape of novel TSSs that can now be studied with this new technology.

## Methods

### RNA preparation

Human lung carcinoma A549 cells (ATCC) were cultured in F12 media supplemented with L-glutamine. Total RNA was purified from cell passage number 3 using the RNeasy RNA purification kit (Qiagen 75142). A260:280 ratio > 2 and RIN > 9.

Human brain total RNA was obtained from Takara (636530). The RNA was isolated by a modified guanidinium thiocyanate method and has RIN > 9.

### ReCappable-seq procedure

Total RNA was (optionally, see text) dephosphorylated using the Quick CIP (NEB M0525; 3 units/ µgRNA at 0.6 units/µL), and the resulting RNA was purified with the “Clean & Concentrate” kit (Zymo Research R1013) using the standard protocol.

Decapping of 5 µg total RNA was performed with 200 units of yDcpS (NEB M0463S) in 10 mM Bis-Tris-HCl (pH 6.5), 1 mM EDTA in 50 µL total volume for 1 h at 37°C. The decapped RNA was purified with the “Clean & Concentrate” kit as above.

Capping with 3′ desthiobiotin GTP (DTB-GTP) was performed in 50 µL total volume with 5 µL VCE (NEB M02080) and 0.5 mM DTB-GTP (NEB N0761), in the absence of SAM for 40 min at 37°C. The RNA was purified with the “Clean & Concentrate 5, Zymo Research R1013” kit and eluted in 40 µL.

The RNA was then fragmented in 0.25× T4 polynucleotide kinase (PNK) buffer (NEB M0201) (1 µL) for 2.5 min at 94°C and immediately put on ice. The solution was further supplemented with 3 µL PNK buffer to 1× and 1 µL of PNK (NEB M0201, 10,000 units/mL) and incubated for 20 min at 37°C. The RNA was purified by AMPure beads in 50% ethanol (1 volume RNA, 2 volumes beads, 3 volumes ethanol). The material was eluted in water, and a portion (20%–25%) was kept as an unenriched control sample. The remaining RNA was mixed with an equal volume of hydrophilic streptavidin magnetic beads (NEB S1421), which had been prewashed two times and resuspended in 2 M NaCl, 10 mM Tris-HCl (pH 7.5), 1 mM EDTA (SA Binding buffer). The suspension was incubated by rotation for 1 h at room temperature. The unbound material was removed by magnetic separation and discarded. The beads containing the bound RNA were washed three times by resuspension in 300 µL of SA binding buffer and three times in 0.25 M NaCl, 10 mM Tris-HCl (pH 7.5), 1 mM EDTA (SA wash buffer). The bound RNA was eluted by incubation of the beads in 0.5 M NaCl, 10 mM Tris-HCl (pH 7.5), 1 mM EDTA containing 1 mM D-biotin (SA elution buffer) by incubating for 30 min at 37°C with occasional resuspension. The eluted material was purified with AMPure beads as above by thoroughly washing the beads and sides of the tube four times with 80% ethanol to eliminate any traces of biotin before proceeding to a second round of streptavidin binding as above.

The eluted material from the second streptavidin purification was purified with AMPure beads as above and the RNA eluted in 32 µL water. This material, in parallel with the unenriched control sample from above, was treated with 4 µL RppH (NEB M0356) in 1× ThermoPol buffer (NEB B90004) in a total volume of 40 µL for 1 h at 37°C. Afterwards RppH was deactivated by adding 0.5 µL 0.5 M EDTA and incubating for 3 min at 94°C. These samples were purified with AMPure beads as above and the RNA eluted in 15 µL of water.

This material was used for generating sequencing libraries using the small RNA library prep set for Illumina (Multiplex compatible) kit (NEB E7330). Typically, 11–13 cycles of PCR were used for the unenriched control RNA and 14–16 cycles for the streptavidin-enriched fraction. Indexed libraries were sequenced using Illumina NextSeq 500 (75-bp paired-end sequencing).

### RT-qPCR procedure

Fifteen micrograms of total RNA from A549 cells was used for the control RT-qPCR experiments after addition of spike-in controls: ERCC mix 1 (0.1 µL per 5 µg RNA) and an in vitro transcription-generated transcript of firefly luciferase (*FLUC*; 5 pg per 5 µg RNA). The mixture was divided into three aliquots, which were subjected to the ReCappable-seq procedure as described above with only one streptavidin enrichment step. One aliquot was first dephosphorylated (CIP sample), a second aliquot was not (no CIP sample), and the third was processed without the decapping step (no DcpS sample). After streptavidin enrichment, all three samples were handled identically.

RNA eluted from streptavidin enrichment and the corresponding unenriched control was purified with AMPure beads, and a portion corresponding to 1 µg input RNA was converted to cDNA using the LunaScript RT supermix cDNA synthesis kit (NEB E3010). One percent of the cDNA was used for each qPCR reaction performed with the Luna universal qPCR mastermix (NEB M3003) and the PCR primers listed (Supplemental Table S2). qPCR reactions were performed in duplicate. The percentage of recovery for each target transcript, for each RNA sample, was calculated from the Cq values of enriched compared with those of unenriched control, which was set to 100% using the following formula: % enrichment = 100 × 2exp − (average Cq input − average Cq SA), where input is the sample before enrichment and SA is the streptavidin enriched sample.

For example, *ACTB* recovery for the “No CIP” RNA sample is calculated as 100 × 2exp − ((25.9,25.7)–(24.2,24.1)) = 32.10%; for the No DcpS sample, 100 × 2exp − ((30.8,29.8)–(20.5,21.0)) = 0.14%.

### RNA-seq

The RNA-seq libraries were prepared as follows: the rRNA was depleted from 0.5 µg of A549 total RNA using the NEBNext rRNA depletion kit (NEB E6310). The resulting rRNA-depleted RNA was used for library construction with the NEB Ultra II directional RNA-seq kit (NEB E7760). The library was sequenced on Illumina MiSeq (75-bp paired-end sequencing). The corresponding sequencing results are referred to as A549 RNA-seq and A549 rRNA-del RNA-seq.

### CAGE

Two replicate samples of total RNA (10 µg per sample) were sent to DNAFORM (Yokohama, Kanagawa, Japan), who performed library preparation and sequencing using the nAnT-iCAGE protocol previously described (Murata et al. 2014). First-strand cDNAs were reverse-transcribed to the 5′ end of capped RNAs and attached to CAGE “bar code” tags, and the sequenced CAGE tags were mapped to the human genome. The indexed libraries were sequenced using Illumina HiSeq 2500 (50-bp single-end sequencing).

### 5′ RACE for RNA Pol III transcripts

We determined the nucleotide sequence of transcript 5′ ends obtained from the amplified RACE products for *RPPH1*, *RMRP,* and *RN7SL1* (Supplemental Fig. S5). Total RNA from A549 cells (3 µg in 30 µL) was treated with RppH (NEB M0356) for 1 h at 30°C and purified by spin column; 0.5 µg of the treated RNA was used for ligation to an RNA oligonucleotide with T4 RNA ligase 1 (NEB M0204) for 1 h at 25°C and then converted to first-strand cDNA with random priming and the ProtoScript II reverse transcriptase mix (NEB E6560). Amplification reactions from this cDNA were performed with the 5′ PCR primer and a reverse primer corresponding to the target sequence using LongAmp Taq polymerase (NEB M0287). The PCR products were sequenced, and the junction between the ligated oligo revealed the start sites.

Sequences of the RNA ligation oligonucleotide and the 5′ forward and reverse primers used are shown in the Supplemental Table S2.

### Bioinformatic analyses

#### Data sets used for TF binding or histone mark analysis in Figure 3 and Supplemental Figures S3 and S4

We used A549 RNA polymerase II ChIP-seq data sets (NCBI Gene Expression Omnibus [GEO; https://www.ncbi.nlm.nih.gov/geo/] accession number GSE33213) for polymerase binding analysis in Figure 3. Because A549 RNA polymerase III ChIP-seq data were not available, we used HeLa Pol III ChIP-seq data sets (GEO accession number GSE20309) (Oler et al. 2010).

For TF and histone ChIP-seq used in Supplemental Figure S3, we downloaded the call sets from the ENCODE portal (Davis et al. 2018; https://www.encodeproject.org/) with the following identifiers: ENCFF103COS ENCFF897HCS ENCFF519XGR ENCFF290AKW ENCFF331NAZ ENCFF447XHR ENCFF603JLN ENCFF538HPA ENCFF473YHH ENCFF000MYX ENCFF387NIL ENCFF721WCM ENCFF359GMW ENCFF623DEN ENCFF931GUX ENCFF000NBM ENCFF897YZE ENCFF102ZYB ENCFF648PQD ENCFF747CVL ENCFF801BLX ENCFF640ILD ENCFF322NBG ENCFF599JTK ENCFF897SFR ENCFF950NVT ENCFF172GSY ENCFF658EZL ENCFF000NDT ENCFF908FKC ENCFF137VHW ENCFF668LIG ENCFF870WJP ENCFF000NEY ENCFF454PBC ENCFF565TWZ ENCFF776IJI ENCFF785XZN ENCFF374NIY ENCFF585RGO ENCFF796BZA ENCFF512ZYH ENCFF997KRD ENCFF967MRB ENCFF829OSK ENCFF063VTN ENCFF000NIP ENCFF967EEA ENCFF539BSO ENCFF604QPX.

For H3K27ac1 and H3K4me1 ChIP-seq used in Supplemental Figure S4, C through E, we used the following ENCODE data: ENCFF640LCC ENCFF872MQN ENCFF312BJX ENCFF874AAY ENCFF189DIW ENCFF843JEO. bigWig files are used as it is. BAM files were compared to the appropriate control BAM files using bamCompare from deepTools (version 3.3.0) (Ramírez et al. 2016; Davis et al. 2018) with the following parameters ‐‐binSize 5 and ‐‐outFileFormat bigWig. FASTQ files were first mapped to GRCh38 using BWA-MEM (version 0.7.12 with default parameters) (Li and Durbin 2009), and the resulting BAM files were processed as described above.

#### K562 CAGE data set

We used the K562 CAGE data set previously generated (Adiconis et al. 2018) for rRNA analysis in Supplemental Figure S2 (GEO accession numbers GSM2772305, GSM2772306, and GSM2772307; three replicates).

#### Genome and annotations

Human genome version GRCh38/hg38 was used for all the analyses.

The comprehensive gene annotation on the primary assembly GRCh38.p5 (GENCODE v24) was downloaded from GENCODE: ftp://ftp.ebi.ac.uk/pub/databases/gencode/Gencode_human/release_24/gencode.v24.primary_assembly.annotation.gtf.gz

To further classify gene types, we added predicted tRNA genes derived from GENCODE v24 tRNAscan into the comprehensive gene annotation for TSS assignment in Figure 4A and Supplemental Figure S6.

The UCSC annotation used for precision and sensitivity assessment was created using the UCSC Table Browser and the following options: assembly, Dec2013 GRCh38/hg38; group, Genes and Gene Predictions; track, All GENCODE v24.

#### Read processing and alignment

Because CAGE reads were 50 nt, we shortened the A549 ReCappable-seq and CIP ReCappable-seq reads to retain the first 50 nt from the 5′ end. Then we processed the ReCappable-seq reads and the paired-end RNA-seq reads by trimming the Illumina adapter using Trim Galore! (version 0.4.4 with parameters ‐‐length 25 ‐‐stringency 3; https://github.com/FelixKrueger/TrimGalore).

Next, we aligned the trimmed reads to the human genome (GRCh38, GENCODE v24) using STAR (Dobin et al. 2013) version 2.5.2, following the ENCODE data processing standard with the following parameters: ‐‐outMultimapperOrder Random, ‐‐outFilterMultimapNmax 20. For ReCappable-seq reads, single-end alignment was used, whereas for RNA-seq reads, paired-end alignment was used. Therefore, local alignment with soft clipping at the 5′end was applied. Only the primary alignments, which were selected using SAMtools (version 1.7 with parameter -F 256) (Li et al. 2009), were used for the TSS analysis and RNA-seq analysis.

#### rRNA analysis

To calculate the percentage of reads from processed rRNA genes, we mapped the ReCappable-seq and CAGE reads from each replicate to a human rRNA reference set (containing rRNA genes 18S, 28S, 5.8S, 12S, 16S) using BWA-MEM (with default parameters) and counted using SAMtools. We did not include the 5S rRNA genes because these genes are transcribed by Pol III polymerase, and therefore, 5S is triphosphorylated.

% rRNA = (number of reads mapped to rRNA genes / number of reads mapped to human genome) × 100.

#### TSS identification and analysis pipeline

The number of 5′ end tags for each position on the genome was calculated using a custom script CountTssGTF.py (https://github.com/elitaone/cappable-seq) and normalized as tags per million primary mappable reads (TPM). Here, 5′ end tag refers to the 5′ most nucleotide of a read mapping to the reference genome, and it corresponds to the TSS position.

To assess reproducibility, we compared the TPM for each position between the two technical replicates starting from the same RNA starting material (Supplemental Fig. S1). Because the two replicates were highly correlated, in order to get more information and to compare between methods for all the ReCappable-seq and CAGE treatments, we merged the aligned reads from two replicates and randomly sampled 63 million primary mappable reads for TSS analysis. In this study, TSS is defined as a position with TPM ≥ 1 (Supplemental Data 1).

To identify high-confidence TSS positions (Fig. 2), we compared the TPM of each TSS position obtained from the ReCappable-seq data set with the corresponding TPM obtained from the unenriched control data set. Positions with an enrichment ratio ≥ 1 were considered as high-confidence TSS (ratio = TPM of ReCappable-seq divided by TPM of unenriched control).

#### Assignment of TSSs to Pol II or non–Pol II

The high-confidence TSSs were further classified by comparing the TPM of each TSS obtained from the untreated ReCappable-seq data set with the corresponding TPM obtained from the CIP-treated ReCappable-seq data set. Based on the TPM Ratio (TPM untreated/TPM CIP-treated), we classified the TSSs into Pol II (TPM Ratio < 4) and non–Pol II (TPM Ratio ≥ 4) (Fig. 2B).

#### Assignment of TSSs to annotated genes in Figure 4A and Supplemental Figure S6

To compare the defined TSSs from both ReCappable-seq and CAGE to GENCODE-annotated genes, including tRNA prediction, for each TSS, we identified the closest exon using BEDTools (Quinlan and Hall 2010) closest with the following options: -t first -D a -iu -s. We assigned a TSS to the corresponding gene if it is within 200 bp upstream or or overlapping with the exon. The number of assigned unique genes or protein-coding genes was counted. Non–Pol II TSSs, not associated with GENCODE-annotated genes or predicted tRNAs, were clustered into individual loci where the TSSs were located within 20 bp of each other.

#### Transcript body coverage in Supplemental Figure S2B

We used a custom script (https://github.com/elitaone/transcript_body_coverage) to analyze the coverage of reads (RNA-seq) or 5′ end tags (ReCappable-seq) across the transcript body. Briefly, we calculated the number of reads or tags covering each position of transcripts >300 bp. For RNA-seq we discarded low expression transcripts (FPKM < 10). For coverage analysis of ReCappable-seq and the corresponding unenriched control, we discarded transcripts whose overlapping 5′end tags contained less than n reads, where n = number of mappable reads divided by 1 million.

#### Enhancer TSSs in Supplemental Figure S4

We used the enhancers annotated in the EnhancerAtlas 2.0 database (Gao and Qian 2019). We matched the forward and reverse ReCappable-seq reads to these enhancers (within a ±5-kb window) and visualized the data using heatmaps. Enhancers that were located distant to first exons (>500 bp) and to other exons (>200 bp) (Andersson et al. 2014; Gao and Qian 2020) in UCSC annotation were overlapped with the Pol II and non–Pol II TSS data sets.

#### Mitochondrial TSSs in Supplemental Figure S9

For TSS identification on mitochondria, we used a stringent standard to filter out the nonunique TSS positions that are from reads also mapping to nuclear chromosomal sites. More specifically, for each TSS position, we calculated the ratio of the number of tags with MAPQ > 39 for Bowtie 2 and MAPQ > 3 for STAR, which indicates unique mapping, respectively, to the number of total tags. Only the positions with Ratio ≥ 0.8 were used as TSS positions plotted on the mitochondrial genome in Supplemental Figure S9A.

#### Precision and sensitivity assessment in Figure 3A and 3B

The assessment of precision and sensitivity was performed as described previously (Adiconis et al. 2018) with minor modifications as shown below.

#### Reference used for precision and accuracy assessment

We mapped our high-confidence A549 ReCappable-seq Pol II TSSs (Fig. 2, quadrant IV) against UCSC annotation, DNase-seq data, and CAGE positions to assess precision. Only the first exon of transcripts included in the UCSC annotation was used for this comparison. The A549 DNase-seq data sets were obtained from ENCODE (bednarrow peak files: ENCFF135JRM, ENCFF698UAH, ENCFF079DJV and ENCFF045PYX, four replicates). The bednarrow peak files from all the replicates were merged for comparison with the ReCappable-seq Pol II TSSs. The A549 RNA-seq data and A549 CAGE data were generated in this study.

#### Calculation of precision and sensitivity

True positives (TPs) were defined as the high-confidence ReCappable-seq Pol II TSSs overlapping each reference, including UCSC TSS annotation, CAGE positions, and DNase-seq peaks as mentioned above. False positives (FPs) were those not overlapping each reference.

False negatives (FNs) were defined as the UCSC TSS annotations that (1) were within any protein coding transcripts with FPKM ≥ cutoff as shown in sensitivity plot *x*-axis (the FPKM was quantified using RSeQC [Wang et al. 2012] based on the A549 RNA-seq data) (Fig. 3B); (2) overlapped at least one A549 DNase-seq peak; and (3) did not overlap any defined high-confidence ReCappable-seq Pol II TSS.

We determined the overlap using BEDTools intersect using the high-confidence ReCappable-seq Pol II TSS as -a and each reference as -b with the addition of the following parameters: (1) -s option for UCSC TSS annotation comparison; (2) -s option for comparison with CAGE positions within a window of ±1 nt.

We calculated precision and sensitivity as shown below:

Precision (Fig. 3A) = TP/(TP + FP) × 100
Sensitivity (Fig. 3B) = TP/(TP + FN)

## Data access

All raw and processed sequencing data generated in this study have been submitted to the NCBI Gene Expression Omnibus (GEO; https://www.ncbi.nlm.nih.gov/geo/) under accession number GSE132660. All custom scripts used for data analysis are uploaded in GitHub (https://github.com/elitaone) and provided in Supplemental Code. The Sanger sequences are available as Supplemental Material. High-confidence TSSs defined by ReCappable-seq have been uploaded as a custom track in the UCSC Genome Browser: http://genome.ucsc.edu/s/rezo/ReCappable-seq. High-confidence TSSs are defined by ReCappable-seq, bedGraph format; negative data values represent CIP-sensitive TSS (quadrant II); positive data values represent CIP-resistant TSSs (Fig. 2, quadrant IV).

## Supplementary Material

Supplemental Material
