# Supplementary material for: Comprehensive determination of transcription start sites derived from all RNA polymerases using ReCappable-seq
Source: Genome Res. 2022 Jan;32(1):162–74. doi: 10.1101/gr.275784.121 (PMC8744680; doi:10.1101/gr.275784.121)
Supplement: Supplemental Material [file supp_32_1_162__DC1.html]

Comprehensive determination of transcription start sites derived from all RNA polymerases using ReCappable-seq — Supplemental Material 

# Comprehensive determination of transcription start sites derived from all RNA polymerases using ReCappable-seq

## Supplemental Material

- Supplemental\_Materials.pdf
- Supplemental\_Tables\_nov\_16.xlsx
- Supplemental\_Data1.txt
- Supplemental\_Code.zip
- Supplemental\_Sequence\_traces.zip
