# Supplementary material for: Comprehensive determination of transcription start sites derived from all RNA polymerases using ReCappable-seq
Source: Genome Res. 2022 Jan;32(1):162–74. doi: 10.1101/gr.275784.121 (PMC8744680; doi:10.1101/gr.275784.121)
Supplement: Supplemental Material [file supp_gr.275784.121_Supplemental_Code.zip › Supplemental_Code.tar.gz!/Users/yan/Documents/NEB/result/RNAseq/Tzertzinis/170927_nextseq/Manuscript_GenomeResearch/finalSubmission/SupplementaryCode/transcript_body_coverage/README_transcriptBodyCoverage.pdf]

README

Author: Bo Yan, yan@neb.com
Revision Date: Sept, 2021

This package is used to calculate and plot the coverage of reads from either directional RNA-seq or 5'end-seq (e.g. CAGE, ReCappable-seq) over eukaryotic transcript body (not including the intron).
See latest updates and codes at: https://github.com/elitaone/transcript_body_coverage

Requirement:
Python 2.7
Samtools and Bedtools preinstalled.
Python packages: numpy, pandas, sklearn.preprocessing, matplotlib, pathos (for multiple processing, not necessary).

Premade files:
All the premade files are available at google drive:
https://drive.google.com/drive/folders/1AOnGNaxhn9VsUuonzMV5Lz7u3uL2bzGR?usp=sharing

Background:
The exon positions of transcripts are divided into N-1 (--number N) bins from 5' to 3' end, which correspond to 1%-100% over the transcript body.
The coverage of each bin is calculated using bedtools multicov considering the strandedness: only the reads or tags aligned to proper strand are counted.
For directional RNA-seq library based on dUTP (e.g. illuminaTruSeq, NEB ultra II RNA-seq), use both Read1 and Read2 for coverage calculation (Read2 is aligned to the RNA strand).
For 5'end-seq, only use Read1 that is aligned to the RNA strand for coverage calculation.
The sum of coverage of each transcript that has FPKM (RNA-seq) or counts of 5'end tags (5' end sequencing) above cutoff is normalized using sklearn.preprocessing.minmax_scale to 0-1 and visualized.

Usage:

1. Use transcript_body_coverage annotation to create an annotation file containing the exon interval information for each transcript.

```
Command to create exon file for counting
$python transcript_body_coverage annotation --input gencode.v24.exon.gtf --output
gencode.v24.exonForcoverage.gtf

Command to generate gtf annotation feature=exon
$awk -v OFS='\t' '{if($3=="exon"){print $0}}' gencode.v24.primary_assembly.annotation.gtf
> gencode.v24.exon.gtf
```

Arguments for transcript_body_coverage annotation

--input: gtf annotation feature=exon

This file is derived from gencode annotation gtf file with feature=exon, using the above awk command.

--output: Exon file used for counting

The output file can be used as the Exon file for transcript_body_coverage count, e.g.

```
chr, source, feature, transcript start, transcript end, transcript id; transcript size, exon 1 start; exon 1 end; exon 2 start; exon 2 end …
chr1    .       .       11869   14409   ENST00000456328.2;1657; +       .       11869;12227;12613;12721;13221;14409;
chr1    .       .       12010   13670   ENST00000450305.2;632;  +       .       12010;12057;12179;12227;12613;12697;12975;13052;13221;13374;13453;13670;
chr1    .       .       14404   29570   ENST00000488147.1;1351; -       .
29534;29570;24738;24891;18268;18366;17915;18061;17606;17742;17233;17368;16858;17055;16607;16765;15796;15947;15005;15038;14404;14501;
```

GTF Format:
column1: chr
column2: source
column3: feature
column4: transcript start
column5: transcript end
column 5: gencode transcript id; transcript size;
column 9: exon 1 start; exon 1 end; exon 2 start; exon 2 end …

2. Use transcript_body_coverage count to count the number of reads overlapping each bin of the transcript, generating a matrix that can be used to plot the coverage.

Only transcripts with length >= max(S, N-1) will be used for coverage calculation. Here S (--size_cutoff) is the transcript size cutoff and N (--number) is number of bins. By default, transcripts with length >= 100 will be used.

For RNAseq, if a FPKM file (--FPKM_file) is provided, N-1 bins are created for transcripts having FPKM >= FPKM_cutoff.
For 5'end-seq or RNAseq without FPKM file, if a Bin file (--bin_gtf) is provided, this Bin file will be used to count the overlapping read. If not provided, a Bin file named Transcript.coverage.bin having N-1 bins will be created based on the Exon file (--gtf). Once generated, this Transcript.coverage.bin file can be used as Bin file for another calculation to speed up the calculation.

Command used to count RNA-seq

```
FPKM file provided:
$python transcript_body_coverage count --type RNAseq --bam RNAseq.primary.bam --output
RNAseq.coverage.report --gtf gencode.v24.exonForcoverage.gtf --FPKM_file RNAseq.FPKM.xls
```

```
FPKM file not provided but Bin file provided:
$python transcript_body_coverage count --type RNAseq --bam RNAseq.primary.bam --output
RNAseq.coverage.report --bin_gtf gencode.v24.exonForcoverage.100bin.gtf
```

Command used to count 5'end-seq

```
Bin file not provided:
$python transcript_body_coverage count --type TSS --bam RecappableSeq.1bp.bam --output
RecappableSeq.coverage.report --gtf gencode.v24.exonForcoverage.gtf
```

```
Bin file provided:
$python transcript_body_coverage count --type TSS --bam RecappableSeq.1bp.bam --output
RecappableSeq.coverage.report --bin_gtf gencode.v24.exonForcoverage.100bin.gtf
```

Arguments for transcript_body_coverage count

--type: TSS or RNAseq
TSS: For 5'end-seq method, e.g. CAGE and ReCappable-seq
RNAseq: For directional RNAseq

--bam:
Bam file with primary mapping, sorted and indexed.

For directional RNA-seq, if bam file contains both Read1 and Read2, both reads are used for coverage calculation considering the strandedness.
For 5'end-seq, bam file is the one base bam file containing only Read1. One base bam file contains 1bp of the most 5' end position, which is generated from bam file using https://github.com/Ettwiller/TSS/bam2firstbasebam.pl.

A premade one base bam file test.onebase.bam and the index file test.onebase.bam.bai are available in the google drive.

--output: Matrix file
A report (tab delimited) containing the number of reads overlapping each bin from 5'->3' end. This report can be used for plotting the coverage using transcript_body_coverage plot.

--gtf: Exon file
An annotation gtf file containing exon information for each transcript. This file can be generated using transcript_body_coverage annotation.
This exon file needs to be provided if the Bin file is not provided.
A premade exon file gencode.v24.exonForcoverage.gtf that is based on gencode Release 24 (GRCh38.p5) is available in the google drive.

--number: number of bins, int N, Default 101
N-1 bins will be created for each transcript for coverage calculation.

--size_cutoff: int S, Default 100
Only transcripts with length ≥ max(S, N-1) will be used for coverage calculation.

--samtools or --bedtools: path of samtools or bedtools, Default available in PATH
If samtools or bedtools is installed and executable in PATH, use default setting and do not need to add these two options.
Otherwise provide the path, e.g. --samtools /miniconda2/envs/my-python2-env/bin/

--bin_gtf: Bin file, Not required

A gtf file containing premade bins for exons of transcripts.

It is not required. But providing this bin file will speed up the calculation.

If provided, this Bin file will be used to count the overlapping read, and the Exon gtf file (--gtf) and the number of bins (--number) will be ignored.
If not provided, a Bin file automatically named Transcript.coverage.bin having N-1 bins will be created under the current directory based on the Exon file (--gtf). This step will take about half an hour to finish.
Once generated, this Transcript.coverage.bin file can be used as Bin file for another calculation to speed up the calculation.

A premade Bin file gencode.v24.exonForcoverage.100bin.gtf, for which all the exons annotated in gencode Release 24 (GRCh38.p5) are split into 100 bins, is available in the google drive.

Positional arguments for RNA-seq

--FPKM_file: FPKM file, Not required

A file generated by RSeQC FPKM_count.py function, which contains the gencode transcript_id (accession in column 4) and FPKM (in last column) as shown below.

```
chr, start, end, accession, mRNA_size, strand, Frag_count, FPM, FPKM
chr1    8352396 8817465 ENST00000337907.7       8026    -       1034    125.433421602   15.6283854475
```

If the FPKM file is not provided, all the transcripts with length ≥ max(S, N-1) will be used to create bins, or use the bins provided in the Bin file if provided.
Provide the FPKM file will speed up the calculation a lot.

--FPKM_cutoff: int, Default 10

Only transcripts with FPKM>=FPKM_cutoff are used for RNA-seq coverage calculation.

3. Use transcript_body_coverage plot to plot one matrix or multiple matrixes.

Plot one or more coverage matrix files generated by transcript_body_coverage count.
The x-axis is the N-1 bins from from 5’ to 3’end corresponding 1%-100% over transcript body.
The y-axis is the normalized coverage for each bin. One or more data can be plotted in the same image.

Command used to plot 5’end-seq enrich and control matrix files

```
$python transcript_body_coverage plot --input D3D4.5end.coverage.matrix
NegD3D4.5end.coverage.matrix --name enrich control --png Endseq.coverage.png --
count_cutoff 65
```

Arguments for transcript_body_coverage plot

--input: Matrix file or files generated by transcript_body_coverage count

--name: names matching the Matrix files

The names should match the Matrix files and will be used as legend to label the results.

If not provided, legends will be labeled 'seq1, seq2….'.

--png: output png file

--Endseq_cutoff: int Nio, Default -1
Only add this option for 5'end-seq to filter low expression transcripts from coverage analysis. Accordingly, only use transcripts having total counts >= Endseq_cutoff for coverage calculation and plotting.
For example, using Nio = number of mappable reads divided by 1 million (e.g. 63 for 63 million reads) equals to applying total TPM of overlapping TSS >= 1 for coverage calculation.
For RNAseq, use default Endseq_cutoff = -1 since low expression transcripts are already filtered based on FPKM_cutoff.

e.g. Output png images of coverage for a. 5'end-seq and b. RNAseq.

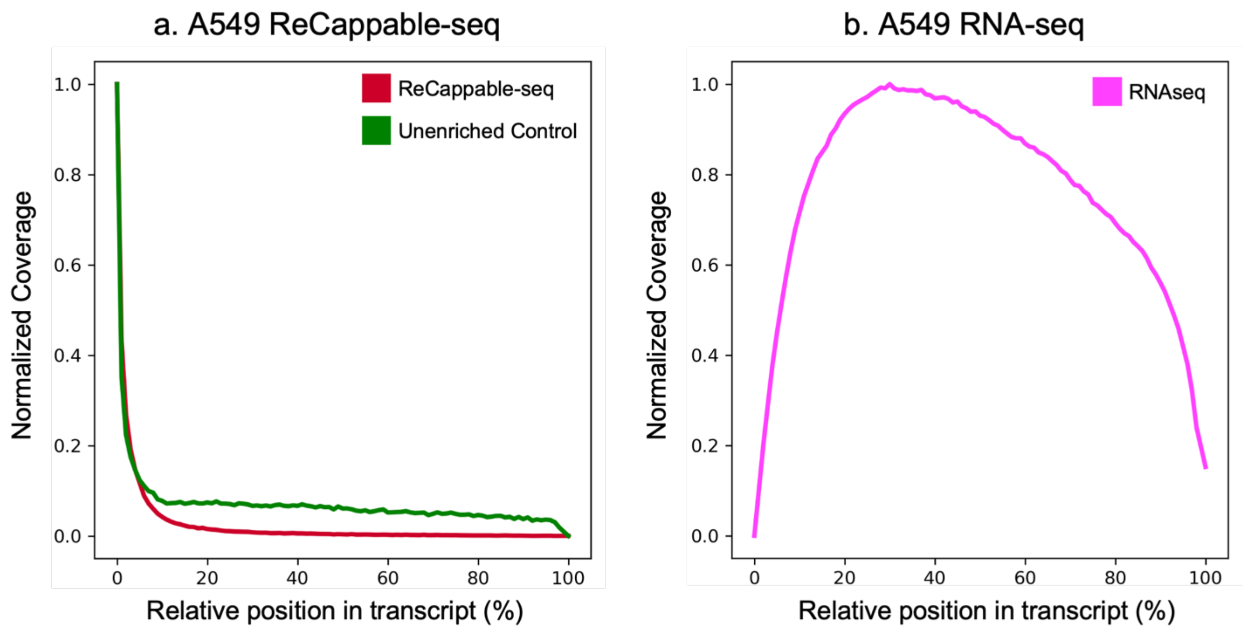

Other notes:

1. Multiple processing on heterogeneous cluster.

This script using pathos module to run multiple processing on heterogeneous computing cluster. If pathos module is not preinstalled, it will try to use the build-in multiprocessing instead, which does not work on heterogeneous cluster. So, without pathos module, it will be slow on heterogeneous cluster.
See https://pypi.org/project/pathos/ for details about pathos module.
